# Viral inactivation of murine coronavirus via multiple gas plasma-derived reactive species

**DOI:** 10.1016/j.redox.2025.103591

**Published:** 2025-03-10

**Authors:** Sander Bekeschus, Meike Heuser, Lea Miebach, Marcus Frank, Thomas von Woedtke, Anke Schmidt

**Affiliations:** a ZIK Plasmatis, Leibniz Institute for Plasma Science and Technology (INP), Leibniz Health Research Alliance, 17489, Greifswald, Germany; b Department of Dermatology and Venerology, Rostock University Medical Center, 18057, Rostock, Germany; c Department of Medical Biology and Electron Microscopy Center, Rostock University Medical Center, 18057, Rostock, Germany; d Institute for Hygiene and Environmental Medicine, Greifswald University Medical Center, 17475, Greifswald, Germany

**Keywords:** CAP, Cold physical plasma, kINPen, Medical gas plasma technology, Murine coronavirus, Reactive oxygen species

## Abstract

The recent pandemic has highlighted the urgent need to elucidate the pathophysiological mechanisms underlying viral effects in humans and is driving the search for innovative antiviral therapies. Several studies have investigated the ability of gas plasma, a partially ionized gas that simultaneously generates several reactive species, to be a new antiviral tool. However, several aspects of the mechanisms of antiviral action of gas plasma remained elusive. In this study, we, for the first time, used a gas plasma device approved for medical purposes and routinely applied in the clinics, especially for wound healing, to test its antiviral activity against a murine corona-virus in vitro (MHV-GFP), a research model analogous to human coronaviruses such as SARS-CoV-2. For this, we established a novel high-content imaging assay that gave quantitative and kinetic information about infection and reduced viral activity in murine fibroblasts (17Cl-1) host cells. Gas plasma treatment delayed viral infectivity and reduced overall infection and toxicity in 17Cl1 cells. Various antioxidants at different concentrations were screened to identify ROS relevant to antiviral effects. Catalase provided no virus protection, and DMSO, mannitol, histidine, Trolox, and ascorbic acid only modestly reduced gas plasma virucidal efficacy. By contrast, glutathione, tyrosine, and cysteine showed profound but not complete protection of MHV from gas plasma-derived reactive species, suggesting pivotal roles of superoxide radicals and peroxynitrite gas in plasma-driven viral inactivation. At extended gas plasma exposure times, fewer intact MHV RNA were detected, indicative of reactive species-driven RNA modifications or degradation as an additional mechanism of action. Virus particle size changes measured by electron microscopy were moderate. Collectively, we identified the potent antiviral activity of a clinically approved argon plasma jet along with potential mechanisms of action.

## Graphical abstract

**Experimental schematic investigating gas plasma-treated murine coronaviruses.** Schematic overview of experiments using the argon plasma jet kINPen for murine coronavirus (MHV) treatment and subsequent infection of murine fibroblasts with MHV followed by subsequent analysis.

**Figure:**
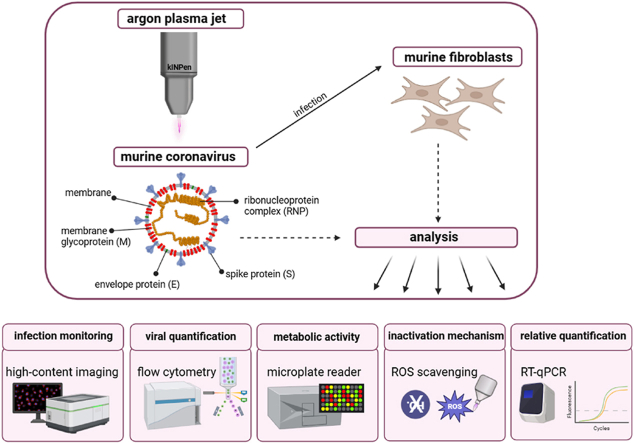

## 1 Introduction

Viral infections continue to pose a significant challenge to global public health. With the COVID-19 pandemic, murine hepatitis virus (MHV) has become particularly relevant as a model for studying SARS-CoV-2 and coronavirus disease [1]. Coronaviruses can cause a variety of clinical manifestations in humans [2], from severe respiratory infections [3], such as viral pneumonia and acute respiratory distress syndrome (ARDS), to significant respiratory distress. Coronavirus infection can lead to myocarditis (inflammation of the heart muscle) and other forms of cardiac dysfunction [4]. In severe cases, coronaviruses can cause thrombotic events [5], neurological symptoms [6], and even multi-organ failure [7]. Here, we used a murine coronavirus model. The MHV-A59 strain causes severe respiratory disease before spreading to other organs [8], similar to the pulmonary pathology observed in COVID-19[9].

Physical antiviral methods include heat (denaturating viral proteins), desiccation, and ultraviolet light. Also, chemical processes (e.g., hydrogen peroxide, H_2_O_2_; alcohol-based disinfectants; sodium hypochlorite, HOCl) enable viral envelope degradation and effectively inactivate viral particles [10], suggesting reactive oxygen and nitrogen species (summarized as ROS) as antiviral effects as previously shown [11,12]. ROS include free radicals such as hydroxyl radicals (OH•) and superoxide anions and non-radical species such as H_2_O_2_, damaging viral proteins, lipids, and nucleic acids to prevent viral replication and infectivity [13]. Medical gas plasma [14], a partially ionized gas generating various ROS, was suggested to have antiviral properties [15,16]. Various gas plasma devices have been shown to reduce viral infections, e.g., herpes simplex virus type 1 [[17], [18], [19], [20]], human adenoviruses [21], and SARS-CoV-2[22].

Understanding the mechanism of action of virus inactivation is critical for the development of effective antiviral treatments. As this is underexplored for gas plasma, we aimed to provide mechanistic insights into the antiviral effects of gas plasma treatment by scavenging different gas plasma-generated ROS. Relative virus particle quantification showed a treatment time-dependent effect, suggesting oxidative damage to the viral envelope, proteins, or nucleic acid, which was partially confirmed by transmission electron microscopy. In addition, we investigated for the first time the antiviral efficacy of an atmospheric pressure plasma device approved for clinical use.

## 2 Experimental section

### 2.1 Cell culture

The murine fibroblast cell line 17Cl-1 was provided by Volker Thiel (Bern University, Switzerland). Cells were cultured in Dulbecco's modified eagle's medium (DMEM) supplemented with 10 % fetal serum (FBS), 1 % l-glutamine, and 1 % penicillin-streptomycin (all PAN Biotech, Germany), and maintained in a cell culture incubator (Binder, Germany) under standard conditions of 37 °C, 95 % humidity, and 5 % CO_2_. Eighteen hours before infection with murine coronavirus (MHV-GFP), 1.5 × 10^4^ cells per well were seeded in 100 μl fully supplemented DMEM containing DAPI (4′,6-diamidino-2-phenylindole; final concentration: 1 μM) in a 96-well flat bottom plate (Thermo Fisher Scientific, Germany). Where indicated, cells were stained with Vybrant DiD (1,1'-dioctadecyl-3,3,3',3'-tetramethylindodicarbocyanine, 4-chlorobenzenesulfonate salt; final concentration 2 μM) cell labeling solution (Thermo Fisher Scientific, Germany) before seeding. Briefly, cells were harvested using accutase cell detachment solution (BioLegend, The Netherlands), washed with PBS (phosphate-buffered saline), and counted for live cells using DAPI and flow cytometry (CytoFLEX S; Beckman-Coulter, Germany). After incubation with 2 μM DID in DMEM for 15 min, the cells were washed with PBS and counted again before seeding.

### 2.2 Viral replication

Recombinant murine coronavirus MHV-A59 with enhanced green fluorescent reporter protein (eGFP) was kindly provided by Volker Thiel (University of Bern, Switzerland). MH virus particles were replicated in 17Cl-1 cells, and virus stocks were prepared by semi-purification using centrifugation steps and three freeze-thaw cycles as described [23]. MHV titers were determined by plaque assay on 17Cl-1 cells and were 2.76 × 10^7^ PFU/ml for all analyses. Briefly, MHV virus stocks were thawed on ice, diluted as required by the experiment in question, added to 96-well plates, supplemented or not supplemented with ROS scavengers, exposed to gas plasma or left untreated, and added (50 μl, approximately 1.38 × 10^6^ of MHV particles) to seeded 17 CL-1 cells per well in another 96-well plate and incubated under standard conditions.

### 2.3 Gas plasma exposure

Gas plasma treatment in this study was performed with the atmospheric pressure argon plasma jet kINPen, similar to the clinical version kINPen MED (neoplas med, Germany) [24]. The jet was operated using argon gas (purity ≥99.999 %; Air Liquide, Germany) at one standard liter per minute flow rate. During gas plasma treatment, the plasma jet was positioned over the 96-well plate using a computer-controlled, motorized xyz stage (CNC, Germany). Viral suspensions were gas-plasma treated in conductive mode as previously described [25]. MHV virus stocks (titer: 2.76 × 10^7^ PFU/ml) were diluted 1:10 in PBS before gas plasma treatment by adding 20 μl viral stock (5.52 × 10^6^ MHV particles) to 180 μl PBS 200 μl per well in a 96-well V-bottom plate (Thermo Fisher Scientific, Germany). Predetermined amounts of double-distilled water were added to compensate for volume loss due to gas plasma-mediated evaporation. Next, virus suspensions were diluted 1:3 in PBS containing 10 % R2F (Roswell Park Memorial Institute 1640 (RPMI1640) supplemented with 2 % fetal calf serum (FCS), 1 % l-glutamine, and 1 % penicillin-streptomycin (all PAN Biotech, Germany)). Finally, 50 μl of each diluted viral suspension was added to each well for infection purposes of cells seeded as described above, corresponding to a multiplicity of infection (MOI) of 3.

### 2.4 Reactive species scavenging and formation in gas plasma-treated liquids

Scavenging of selected reactive species was achieved with the following substances: ascorbic acid (aa; 0.01 mM, 0.1 mM, 1 mM), catalase (cat; 5 μM), cysteine (cys; 0.1 mM, 1 mM, 10 mM), glutathione (GSH; 0.5 mM, 5 mM, 50 mM), histidine (his; 0.1 mM, 1 mM, 10 mM), mannitol (2 mM, 10 mM, 50 mM), N-acetylcysteine (NAC; 1 mM, 10 mM, 20 mM) (all Sigma Aldrich, Germany), and dimethyl sulfoxide (DMSO; 0.1 mM, 1 mM, 10 mM; Carl Roth, Germany). For experiments, 200 μl per well, consisting of 20 μl virus suspension, scavenger, and PBS, were gas plasma-treated. Reactive species formation in liquids (PBS) was evaluated using fluorescent and colorimetric sensors. Briefly, 100 μl PBS was exposed to gas plasma in presence or absence of cPTIO (2-(4-Carboxyphenyl)-4,4,5,5-tetramethylimidazoline-1-oxyl-3-oxide potassium salt, 100 μM; Thermo Fisher, Germany) as a scavenger for nitric oxide (NO^.^). Aminophenyl fluorescein (APF) and hydroxyphenyl fluorescein (HPF) (both Thermo Scientific, Germany) were used to assess peroxynitrite (ONOO^−^), hydroxyl radical (^.^OH), and/or hypochlorous acid (HOCl) deposition as described before [26]. Immediately after gas plasma treatment, the fluorescence of the dyes in liquid was determined at *λ*_ex_ 485 nm and *λ*_em_ 525 nm using a microplate reader (Infinite F200 Pro; Tecan, Switzerland).

### 2.5 Metabolic activity

The metabolic activity of 17Cl-1 cells was assessed 24 h after virus infection using resazurin (15,5 μl of 7-hydroxy-3H-phenoxazin-3-on-10-oxid, 100 μM; Alfa Aesar, Germany). Resazurin is reduced by metabolically active cells to fluorescent resorufin, which was quantified at *λ*_ex_ 530 nm and *λ*_em_ 590 nm using a microplate reader (Infinite M200 pro; Tecan, Switzerland). In kinetics, measurements were performed at 20 min-intervals for 1–5 h after adding resazurin, while cells were maintained at 37 °C and 5 % CO_2_ in the microplate reader.

### 2.6 High-content imaging

Monitoring of MHV-infected 17Cl-1 cells was performed using high-content imaging (Operetta CLS; PerkinElmer, Germany). Images were acquired at 1 h-intervals for up to 24 h (in some cases 72 h) after virus infection under life-cell imaging conditions (37 °C and 5 % CO_2_) using a 1.25 × air (NA 0.03) objective (Zeiss, Germany). Acquisition channels used were brightfield (BF) and fluorescence channels (bandwidth center) at *λ*_ex_ 365 nm and *λ*_em_ 465 nm for DAPI, *λ*_ex_ 475 nm and *λ*_em_ 525 nm for GFP, and *λ*_ex_ 630 nm and *λ*_em_ 680 nm for DiD. Cell viability (i.e., DAPI-negative cells), cell confluence (i.e., DiD-positive area), and viral particle distribution (i.e., GFP-positive area) of MHV-infected 17Cl-1 cells were determined using algorithm-driven quantitative image analysis using the Harmony 4.9. software (PerkinElmer, Germany). Whole-well imaging was performed, covering the entire area (32 mm^2^) of each 96-well plate well.

### 2.7 Analysis of viral particle values after gas plasma treatment using one-step qPCR

Viral RNA was extracted using the QIAmp viral kit according to the manufacturer's instructions (Qiagen, Germany). Total RNA was stored at −80 °C and subjected to one-step qPCR analysis after concentration measurements. qPCR detection of viral targets was performed using the RealTime ready RNA virus master (Roche, Germany). The MHV qPCR mix contained primers against the conserved M gene sequence in the MHV-A59 strain [8] (5`-GGAACTTCTCGTTGGGCATTATACT-3` as forward primer and 5`- ACCACAAGATTATCATTTTCACAACATA-3` as reverse primer, and single-color probe 5`-FAM-ACATGCTACGGCTCGTGTAACCGAACTGT-BHQ1-3′) used at a final concentration of 0.4 μM. Alternatively, direct RT-qPCR detection of gas plasma-treated and untreated MHV (without prior RNA isolation) was performed using the Luna Universal One-Step RT-qPCR Kit (NEB, Germany). For this, a thermolysis step of MHV particles was performed at 70, 80, 85, 90, and 95 °C for 5 min or 10 min (adapted from Ref. [27]) in a thermocycler (Biometra, Germany). As samples, virus stocks were diluted 1:10 in PBS (treatment volume 200 μl) and treated with gas plasma for 10 s, 30 s, 60 s, and 120 s, and different concentrations of viral suspension were added to the reaction mix. The PCR protocol was as follows: 50 °C for 8 min for reverse transcription; 60 s at 95 °C for denaturation, 10 s at 95 °C and 30 s at 60 °C for extension. The RT-PCR reaction was carried out for a total of 40 cycles [27]. Cycle threshold (C_T_) values were measured and compared with each gas plasma treatment.

### 2.8 Electron microscopy of MHV particles

MHV suspension (max. 3 ml) was dialyzed against PBS overnight at 4 °C to obtain purified MHV particles. Slide-A-Lyzer G3 dialysis cassettes (2K MWCO, FisherScientific, Germany) were used. Dialyzed MHV were gas plasma-treated for 60 s or were left untreated and immediately supplemented with paraformaldehyde (PFA) and glutaraldehyde (GA) so that a final concentration of at least 2 % of the former and 0.1 % of the latter was achieved. For transmission electron microscopy (TEM) analysis using the negative staining method, the samples were adhered to 400 mesh copper TEM grids with formvar film coated with carbon (Plano GmbH, Wetzlar Germany). Before sample incubation, the grids were pre-treated with a 1 % aqueous solution of alcian blue for 10 min. After brief washing with distilled water, the grids were placed on 30 μl drops of the viral suspension for 20 min, followed by washes and a final contrasting step using a 1 % aqueous solution of uranyl-acetate. After drying, the grids were examined using a Zeiss EM902 transmission electron microscope (Zeiss Microscopy, Oberkochen, Germany) operated at 50 kV. Digital images were acquired using a 1 × 2k FT-CCD camera (TRS; Proscan, Scheuring, Germany) using iTEM camera control and imaging software (Olympus Soft Imaging Solutions, Münster, Germany). Subsequently, some data was processed with Photoshop CS6 (Adobe, USA) to analyze, e.g., median particle sizes.

### 2.9 Statistical analysis

Graphing and statistical analysis were performed using Prism 9.5.1 (GraphPad Software, USA). Unless otherwise noted, data are presented as mean ± standard deviation (S.D.). Statistical analysis was performed using *t*-test for comparison between two groups or one-way analysis of variance (ANOVA) for comparison between multiple groups, as indicated in the figure legends. Experiments were repeated at least three times per condition. Data were summarized by determining the area under the curve (AUC) for fluorescence kinetic experiments (e.g., GFP expression). Significance levels are indicated as follows: ∗p < 0.05, ∗∗p < 0.01, ∗∗∗p < 0.001.

## 3 Results

### 3.1 Establishing a sensitive and kinetic MHV infection high-content imaging method

**Fig. 1:**
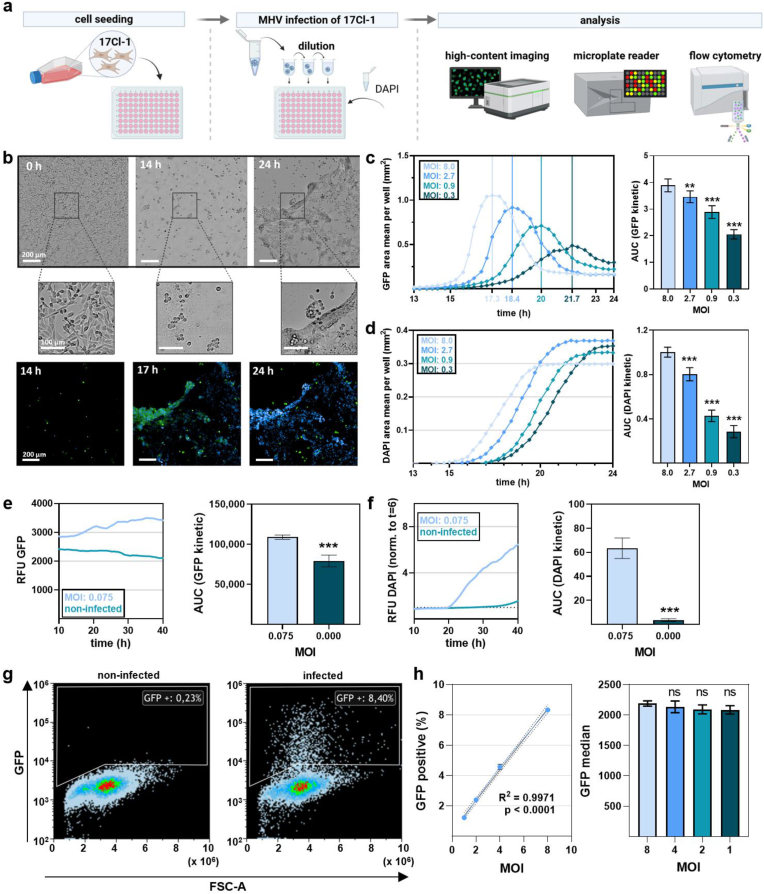
**Monitoring MHV infection in 17Cl-1 cells.** (**a**) Schematic overview of infection experiments using murine fibroblasts (17Cl-1) with different dilutions of murine coronavirus (MHV) followed by subsequent analysis; (**b**) representative images of 17Cl-1 fibroblasts at different time points after infection with MHV with images illustrating cytopathic effects of MHV infection in 17Cl-1 (top panel and higher magnification inserts in the middle panel) in bright field and fluorescence (DAPI, blue; GFP, green; bottom panel); (**c**) algorithm-driven image analysis of GFP area over time in infected 17Cl-1 cells at different MOIs with vertical lines representing maximum areas per MHV dilution (left) and corresponding AUCs from kinetics (right); (**d**) algorithm-driven image analysis of dead (DAPI^+^) cells (left) at different MOIs, and corresponding AUCs from kinetics (right); (**e**) relative fluorescence units (RFU) of GFP intensity in infected cells kinetically (left) and corresponding AUCs (right) as measured in a microplate reader; (**f**) DAPI RFU of dead infected cells kinetically (left) and corresponding AUCs (right) as measured in a microplate reader; (**g**) representative flow cytometry density plots of MHV-GFP infected and non-infected 17Cl-1 cells, showing forward scatter (FSC) against fluorescent GFP signal indicative of infected cells; (**h**) flow cytometry quantification of GFP-positive cells (% left) and average GFP intensity per GPF-positive cell (right). Data are presented as mean ± S.D. of a representative of three experiments. Statistical analysis was performed using anova (**∗**p < 0.05; ∗∗p < 0.01; ∗∗∗p < 0.001). MOI = multiplicity of infection. AUC = area under the curve. GFP = green fluorescent protein. DAPI = 4′,6-diamidino-2-phenylindole. Scale bars are 200 μm, and scale bars of inserts are 100 μm.

**Fig. 2:**
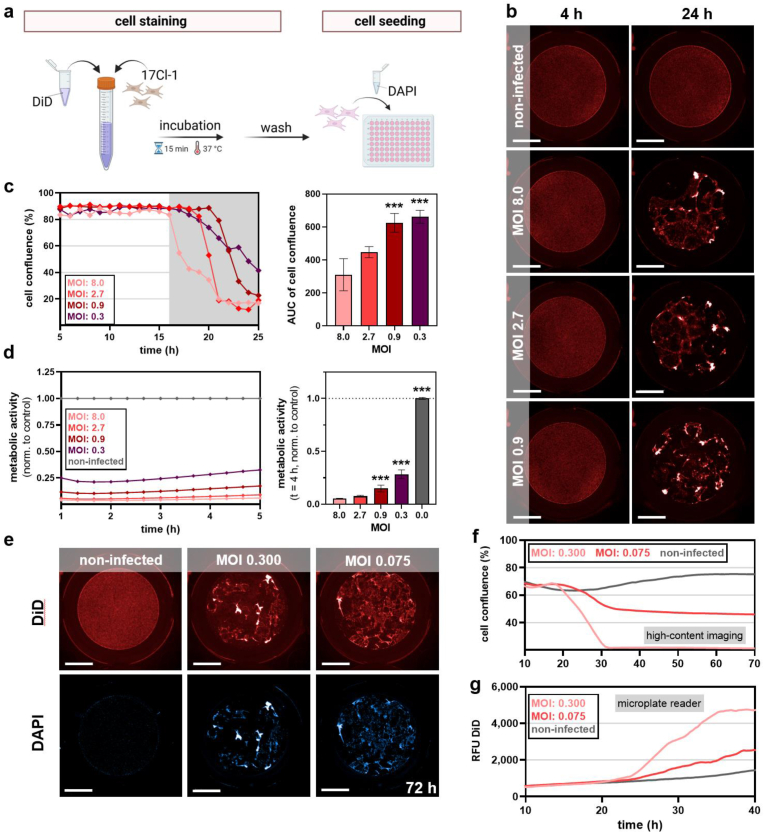
**Cell labeling to identify cytopathic MHV effects in 17Cl-1 cells kinetically.** (**a**) Schematic overview of cell staining and seeding procedure, including DiD and DAPI staining of 17Cl-1 cells; (**b**) representative images showing the cytopathic MHV effect on DiD-stained 17Cl-1 cells at different MOIs and time points; (**c**) algorithm-driven image analysis of DiD-based cell confluence 5–24 h after MHV infection assayed kinetically (left) and corresponding AUCs (16h–24h; right); (**d**) metabolic activity assayed via resazurin of 17Cl1 cells 24 h after MHV infection assayed kinetically (left) and corresponding AUCs (24h–28h; right); (**e**) representative images showing the cytopathic effect on DiD and DAPI--stained cells 72 h after MHV infection; (**f**) algorithm-driven quantitative image analysis of DiD-based cell confluence 10–70 h after MHV infection; (**g**) relative fluorescence units (RFU) of DiD-labeled 17Cl-1 cells 10–40 h after MHV infection as assessed via microplate reader. Data are presented as mean ± S.D. of a representative of three experiments. Statistical analysis was performed using anova (∗∗∗p < 0.001). Scale bars are 2 mm. MOI = multiplicity of infection. AUC = area under the curve.

The murine coronavirus strain MHV-A59 (murine hepatitis virus A59), which expresses enhanced green fluorescence protein (eGFP), was used to monitor infection of 17Cl-1 murine fibroblasts and their analysis (Fig. 1a). At 14 h post infection, fusion of multiple cells to multinucleated cell bodies (syncitia) as cytopathic effect (CPE) was observed followed by monolayer disruption of infected (GFP-positive) and terminally dead (DAPI-positive) cells (Fig. 1b). The higher the MOIs (multiplicity of infection) used for infection, the earlier the maximum GFP area and the larger area under the curve (AUC) of the infection kinetic was (Fig. 1c). For terminally dead cells, results were similar, showing an earlier rise of total DAPI-positive areas at higher MOIs (Fig. 1d). For comparison, the same experiments were also performed for one MOI and more extended kinetics in a microplate reader, which gave results with lower resolution for GFP^+^ MHV infected over non-infected cells (Fig. 1e) while DAPI signals resolved appropriately (Fig. 1f). To validate infection on a single-cell level with high sensitivity, flow cytometry was used, showing a 17Cl-1 population with increased fluorescence in MHV infected cells (Fig. 1g). A clear relationship between MOI and the percentage of GFP-positive cells was found, while the degree of infectivity was similar regardless the MOI as suggested by similar median fluorescent intensities (Fig. 1h). These results confirmed the ability in our model to monitor MHV-infection in a time-resolved scale. However, viral action is usually monitored by plaque formation in cellular monolayers. This led us to establish a method of dynamically monitoring cellular growth area (confluence, the inverse area of plaque formation) in fluorescently labeled 17Cl-1 cells (Fig. 2a) to analyze MHV infectivity (Fig. 2b). The assay was highly sensitive to resolve the cellular confluence at different MOIs kinetically and expressed as AUCs (Fig. 2c). As a more terminal read out, analyzing 17Cl-1 infection by metabolic activity kinetically assessed at 24 h in the microplate reader confirmed the overall reduced cell activity at higher MOIs mostly independent of resazurin incubation time (Fig. 2d). To exclude late-stage infections, high-content imaging monitoring for up to 72 h post infection (Fig. 2e) revealed tracking of the first 20–30 h to be appropriately sensitive for analysis (Fig. 2f), which was confirmed by long-term microplate reader experiments (Fig. 2g). Collectively, these results indicate the excellent sensitivity and resolution of our high-content imaging analysis method to monitor MH virus infection in 17Cl-1 cells.

### 3.2 kINPen argon gas plasmas markedly reduced MHV infectivity

**Fig. 3:**
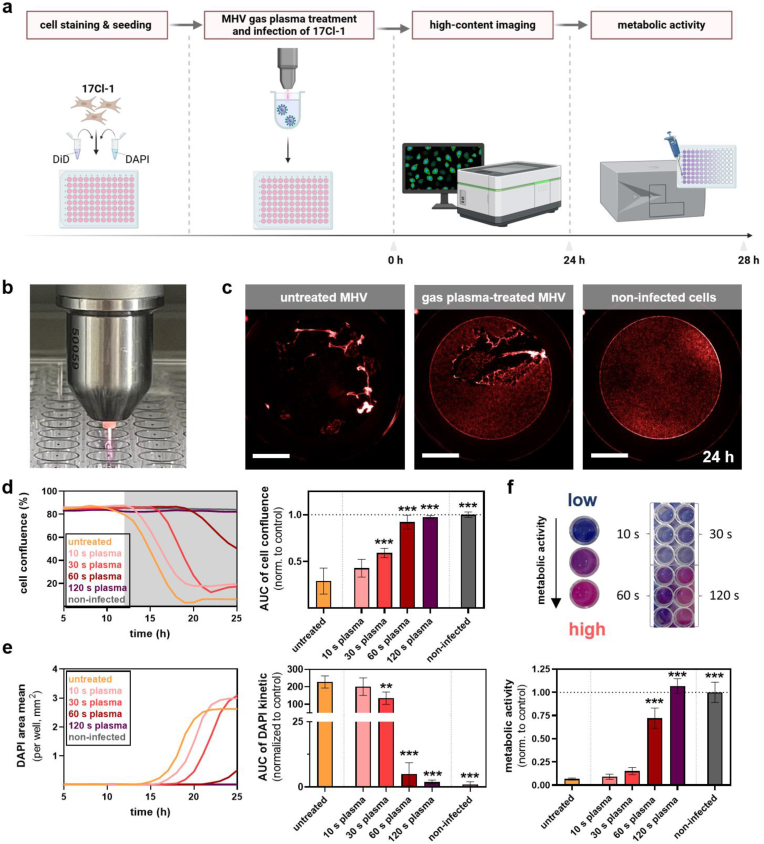
**Antiviral activity of MHV gas plasma treatment.** (**a**) Schematic overview of experimental workflow; (**b**) representative image of atmospheric pressure argon plasma jet kINPen treatment of MHV virus solutions in a 96-well plate; (**c**) representative images showing the partially abolished cytopathic effect in 17Cl-1 cells within gas plasma-treated MHV infection experiments; (**d-f**) algorithm-driven image analysis of DiD-based cell confluence (**d**) and terminally dead cells (**e**) after infection with untreated or gas plasma-treated MHV with corresponding cell confluence (**d**) and DAPI (**e**) AUCs; (**f**) representative images of resazurin metabolic activity assay (top) quantification of metabolic activity in cells infected with untreated or gas plasma-treated MHV after 28 h (bottom). Data are presented as mean ± S.D. of a representative of three experiments. Statistical analysis was performed using anova with untreated MHV as comparator (∗∗p < 0.01; ∗∗∗p < 0.001). Scale bars are 2 mm. AUC = area under the curve.

We next exposed MHV suspensions to gas plasma at various treatment times before adding virus particles to 17Cl-1 experiments and kinetic monitoring infection (Fig. 3a). For this, the atmospheric pressure argon plasma jet kINPenwas used for experiments (Fig. 3b) and demonstrated a marked reduction of plaques in the 17Cl-1 cell layer with gas plasma-treated MHV compared to untreated MHV particles (Fig. 3c). Inactivation of MHV infectivity followed a treatment time-dependent response of gas plasma exposure in terms of abrogating loss of MHV-mediated cellular confluence (Fig. 3d) as well as the area with terminally dead cells (Fig. 3e). Significant MHV inactivation was achieved with 30 s of kINPen plasma exposure. For metabolic activity analysis, significant protection of MHV-induced metabolic activity reduction 24 h post-infection was achieved for 60 s of kINPen plasma exposure (Fig. 3f). Gas plasma-treated PBS, in which the virus particles were contained, retains some of the oxidant, which can confer cytotoxic effects [28]. To exclude such effects, PBS in the absence of MHV was gas plasma-treated and added to 17Cl-1 cells, and their activity was analyzed. A strong impact of gas plasma-treated PBS to reduce the metabolic activity in 17Cl-1 cells was not observed (Fig. S1a). Likewise, to exclude the impact of the argon gas alone to affect MHV infectivity, MHV suspensions were exposed to this noble gas for different durations, and no change in mediating reduced metabolic activity in 17Cl-1 cells was observed (Fig. S1b). Altogether, these data suggested kINPen gas plasma treatment affecting the ability of MHV to enter and/or replicate within 17Cl-1 cells effectively.

### 3.3 ROS scavengers indicated gas plasma species responsible for virus inactivation

**Fig. 4:**
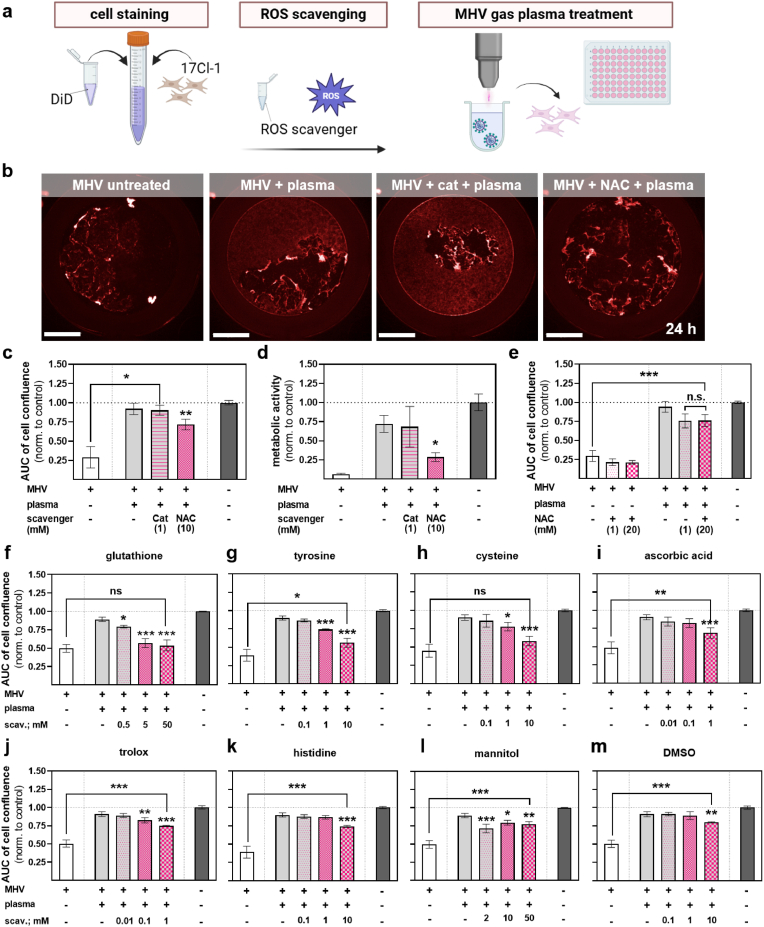
**Amplitude of ROS scavengers protecting MHV from gas plasma-mediated inactivation.** (**a**) Schematic overview; (**b**) representative images showing the impact of untreated and 60 s plasma-treated MHV with or without prior NAC or catalase addition to DiD-stained 17Cl-1 cells 24 h after start of infection; (**c-d**) algorithm-driven quantitative image analysis and corresponding AUC of 17Cl-1 cell confluence 12–24 h after MHV infection with viruses being untreated or gas plasma treated in the presence or absence of catalase or NAC (**c**) and single time point metabolic activity of the cells (**d**); (**e**) relative cell confluence at different NAC concentrations; (**f-m**) relative 17Cl-1 cell confluence in untreated or gas plasma-treated MHV infection experiments in the presence or absence of various scavengers and concentrations added before gas plasma treatment, i.e., glutathione (**f**), tyrosine (**g**), cysteine (**h**), ascorbic acid (**i**), Trolox (**j**), histidine (**k**), mannitol (**l**), and dimethyl sulfoxide (**m**). Data are presented as mean ± S.D. of a representative of three representative experiments. Statistical analysis was performed using anova (∗p < 0.05; ∗∗p < 0.01; ∗∗∗p < 0.001). Scale bars are 2 mm. AUC = area under the curve; NAC = N-Acetylcysteine; cat = catalase; DMSO = dimethyl sulfoxide.

**Fig. 5:**
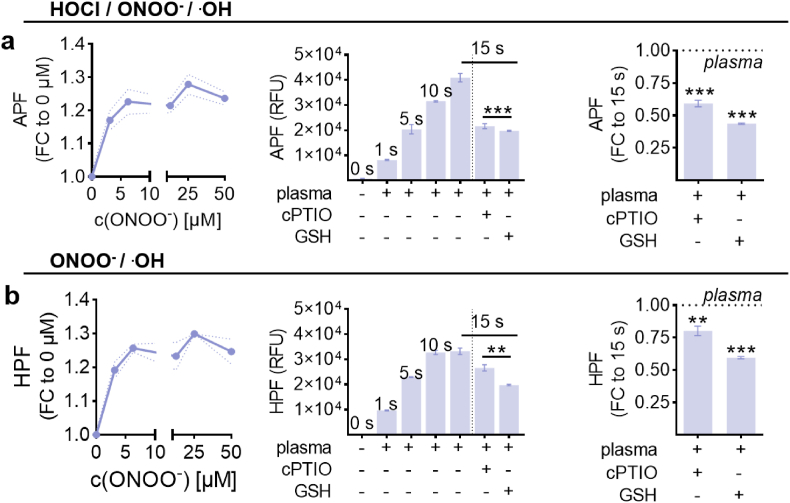
**Use of fluorescent probes as an indicator for peroxynitrite production.** (**a-b**) APF (**a**) and HPF (**b**) fluorescent intensity for different ONOO^−^ concentrations and gas plasma treatment as measured using a microplate reader. Data are mean + S.D., and statistical analysis was performed using anova (∗∗∗p < 0.001).

To next identify the reactive species produced from the kINPen gas plasma playing a critical role in reducing MHV infectivity, we used our inverse plaque assay to screen 10 different ROS scavengers or enzymes for their ability to shunt gas plasma-mediated MHV inactivation (Fig. 4a). For this, we used 60 s of gas plasma exposure time as this consistently reduced MHV-mediated plaque formation (Fig. S1c) while being not too long to inactivate all viruses (Fig. 4b) and potentially consume the antioxidants tested, and none of the antioxidants showed effects on the growing cultures of untreated and uninfected 17Cl-1 cells (Fig. S3). Hydrogen peroxide (H_2_O_2_) is a known primary mediator of gas plasma-mediated toxicity in eukaryotic cells [29]. Interestingly, H_2_O_2_ removal via catalase (cat) did not protect MHV from gas plasma-mediated inactivation in terms of cellular confluence (Fig. 4b and c) or metabolic activity (Fig. 4d). N-acetylcysteine (NAC) had good protection in the latter and some protection in the former assay (Fig. 4b). To exclude potential effects on NAC consumption, two concentrations (1 mM and 20 mM) were tested. Both gave similar results (Fig. 4e), indicating the only limited ability of NAC to protect from gas plasma-mediated MHV inactivation. A strong protection effect was observed for glutathione (GSH), which was concentration-dependent (Fig. 4f). Such findings were also made for the antioxidant amino acids tyrosine (tyr, Fig. 4g) and cysteine (cys, Fig. 4h). To a lesser extent, but in a concentration-dependent manner, reduction of gas plasma-mediated MHV inactivation was also observed for ascorbic acid (aa, Fig. 4i), vitamin E (Trolox, Fig. 4j), and histidine (his, Fig. 4k). Mannitol (Fig. 4l) and dimethyl sulfoxide (DMSO, Fig. 4m) provided significant but modest protection of MHV particles from gas plasma-derived reactive species to. Relative reduction rates of these compounds on gas plasma-mediated inactivation of MHV infectivity attributed the highest effect to GSH, tyrosine, and cysteine (Table 1). GSH scavenges superoxide radicals, which is also the case for the less effective histidine. GSH also scavenges hydroxyl radicals similar to the less effective mannitol and DMSO. Finally, GSH scavenges H_2_O_2_, analogously to the non-effective protection by catalase. Thus, organic peroxyl radicals and peroxynitrite (ONOO^−^) remained as possible agents of gas plasma-mediated MHV inactivation. Results using APF and HPF, with the latter shown to be sensitive to ONOO^−^ and hydroxyl radicals and the former additional to hypochlorous acid [26], indicated probe protection with nitric oxide scavenger cPTIO, especially for APF (Fig. 5a) over HPF (Fig. 5b) (right panels). Also, compared to experimentally added ONOO^−^ exposure of the probes (left panels), lower fold-changes in fluorescence were increased as compared to gas plasma exposure (middle panel), suggesting ONOO^−^ to be introduced to gas plasma-treated liquids, which could have contributed to the antiviral effects observed.

**Table 1:** **Reactive species scavengers and enzymes**. Several scavengers (e.g., N-acetylcysteine, catalase, glutathione, tyrosine, cysteine, ascorbic acid, Trolox, histidine, mannitol, and dimethyl sulfoxide) were used to reduce or abolish gas plasma-derived reactive species. The differences in area under the curve (AUC) (%) were determined for gas plasma-treated MHV plus scavenger and compared to gas plasma-treated MHV alone. NAC = N-acetylcysteine; cat = catalase; DMSO = dimethyl sulfoxide; H_2_O_2_ hydrogen peroxide; •OH = hydrogen radical; HOCl = hypocaloric acid; RO• = alkoxyl radical; ROO• = peroxyl radical; ONOO^−^ = peroxynitrite; O_2_^−^• = superoxide anion; ^1^O_2_ = singlet oxygen; NO_2_• = nitrogen dioxide radical.

scavenger	concentration used (mM)	reported to scavenge the following reactive species	difference in area under the curve (AUC) to gas plasma exposure (%)
glutathione	5.0	^1^O_2_, •OH, ROO•, H_2_O_2_, ONOO^−^	35.6
tyrosine	10	H_2_O_2_, O_2_^•−^, ONOO^−^, •OH	33.8
cysteine	10	H_2_O_2_, O_2_^•−^	31.9
ascorbic acid	1.0	•OH, H_2_O_2_, NO_2_•, O_2_^•−^,^1^O_2_	21.8
NAC	10	•OH, HOCl, H_2_O_2_	20.3
Trolox	1.0	•OH, RO•, ROO•	16.3
histidine	10	^1^O_2_, •OH	15.6
mannitol	50	•OH	12.0
DMSO	10	•OH	11.0
catalase	1.0	H_2_O_2_	1.92

### 3.4 MHV RNA quantification as an indicator of integrity after gas plasma exposure

**Fig. 6:**
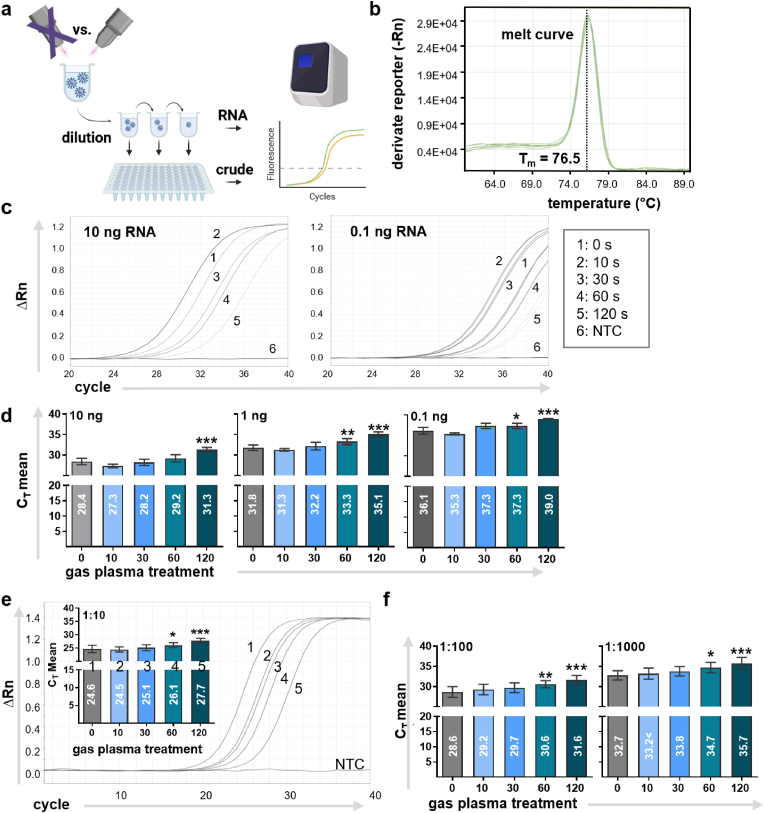
**mRNA analysis in gas plasma-treated MHV particles.** (**a**) Schematic overview of one-step RT-qPCR where viral nucleic acids were assayed either after RNA isolation or without previous RNA isolation (crude viral suspension) in a one-step reaction; (**b**) single sharp peak at T_m_ = 76.5 °C indicating a specific amplification of target RNA; (**c**) qPCR amplification curves of gas plasma-treated viral RNA of 10 ng or 0.1 ng exposed for 10 s, 30 s, 60 s, and 120 s compared to untreated viral RNA; (**d**) C_T_ values of various MHV RNA concentrations with different gas plasma treatment times of MHV particles followed by RNA extraction and one-step RT-qPCR; (**e**) qPCR amplification curves and C_T_ values across various gas plasma treatment times analyzed at 1:10 MHV particle dilution; (**f**) C_T_ values for two additional MHV particle dilutions (1:100 and 1:1000). Data are presented as mean ± S.D. of three experiments. Statistical analysis was performed using anova (∗p < 0.05; ∗∗p < 0.01; ∗∗∗p < 0.001). C_T_ = cycle threshold; NTC = non-target control.

We next addressed the question of whether gas plasma exposure potentially degraded intra-particle MHV RNA, ultimately contributing to reduced infectivity as a potential mechanism of action. To quantify and monitor cycle threshold (C_T_) values indicative of the amount of RNA bound to primers, we used both dialyzed crude MHV suspensions and isolated MHV-derived RNA in a one-step RT-qPCR. MHV particles could be detected either directly after a thermolysis step of the (crude) MHV suspension or after an additional RNA isolation step (Fig. 6a). First, a single and defined melting point (Tm = 76.5 °C) indicated that the amplified qPCR product was homogeneous, specific, and highly enriched (pure) with the MHV primers used, i.e., no shoulder peaks were observed (Fig. 6b). Next, crude viral suspensions were dialyzed in PBS overnight. The efficiency of RNA isolation was evaluated by varying the RNA concentration to determine the optimal conditions for one-step RT-qPCR. The concentrations between 0.01 and 100 ng showed C_T_ values in the range of 28–39 (Fig. S4a), while higher RNA concentrations did not markedly enhance C_T_ values (Fig. S4b). The lowest RNA concentration of MHV particles that resulted in an acceptable C_T_ value (28) was 10 ng/μl (Fig. 6c). Next, gas plasma-treated MHV suspension was compared to untreated MHV particles to detect potential effects on viral RNA. After isolating viral RNA, we used RNA concentrations ranging from 10 ng to 0.1 ng for PCRs and all gas plasma treatment times. Interestingly, we found significantly higher C_T_ values for long-term gas plasma exposure of MHV particles (Fig. 6d), suggesting a direct, possibly degrading effect of gas plasma on MHV RNA.

To directly use viral MHV particles as an RT-qPCR template, bypassing the time-consuming RNA isolation step, and to evaluate direct gas plasma effects on MHV, we also applied a one-step RT-qPCR protocol using dialyzed crude MHV suspensions. Our question was whether additional thermolysis is necessary for viral membrane lysis. Therefore, the dependence of the C_T_ values on the thermolysis of dialyzed viral particles at a 1:10 dilution was investigated by varying the temperature between 70, 80, 85, 90, and 95 °C and two exposure times of 5 min or 10 min. By analyzing the C_T_ values using qPCR, we obtained slight changes in the C_T_ value depending on the thermolysis condition but no clear tendency of the C_T_ value-thermolysis relationship was observed (Fig. S4c). Other methods of disaggregating MHV, such as ultrasound or three freeze-thaw cycles, showed no change in C_T_ values (data not shown). Due to the minor influence of an additional thermolysis step, qPCR was performed without this step and yielded solid C_T_ values over several dialyzed, untreated MHV particle dilutions (Fig. S4d). However, when viral RNA is used without an additional dialysis step of the MHV suspension, RT-qPCR does not allow precise amplification. Therefore, no meaningful correlation of the C_T_ value with different RNA concentrations was observed (Fig. S4e). This result highlighted the importance of using a dialyzed MHV starting suspension for RNA isolation. Nevertheless, we were able to replicate the results found with dialized MHV when using undialized crude MHV (without RNA isolation), showing significantly increased C_T_ values with increasing gas plasma exposure times for low (Fig. 6e) and high (Fig. 6f) virus particle dilutions.

### 3.5 Structural analysis of viral particles using electron microscopy

**Fig. 7:**
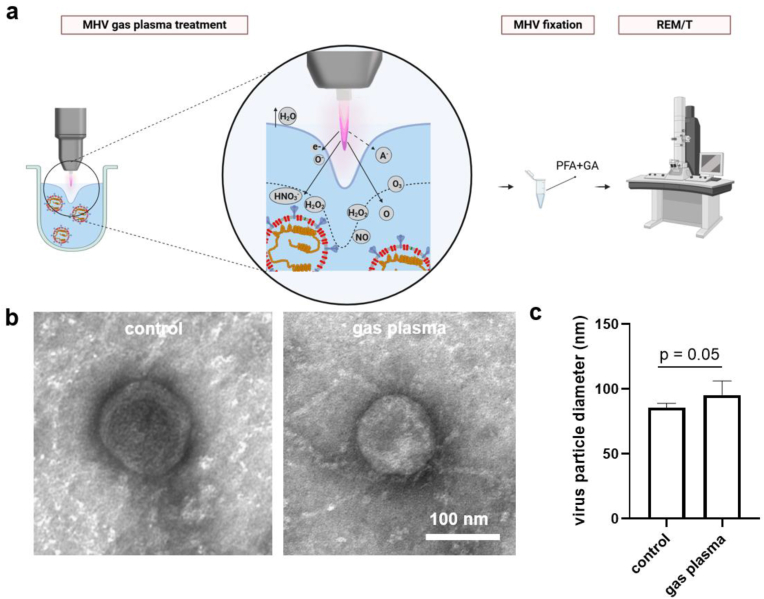
**Ultrastructure electron microscopy analysis of gas plasma-treated MHV.** (**a**) schematic overview of the TEM procedure using dialyzed MHV being untreated or gas plasma-treated and fixed in PFA (7 %) and GA (0.1 %); (**b**) representative cryo-EM images without (left) and with gas plasma treatment (right); (**c**) quantitative size (membrane-to-membrane diameter) of 5–8 MHV particles per group. Scale bars represent 200 nm and 100 nm. PFA = paraformaldehyde; GA = glutaraldehyde.

For examination of general MHV characteristics such as shape, size, and envelope features (e.g., thickness) after gas plasma treatment, transmission electron microscopy was employed (Fig. 7a). For this, dialyzed and non-dialyzed particles were exposed to gas plasma for 60s, followed by fixation in paraformaldehyde (PFA) and glutaraldehyde (GA) and subsequent electron microscopy analysis. Challenges in the preparation process only allowed imaging of a small sample number of particles. In particular, for 50kX magnification, two-digit numbers of virus particles could be observed. To have a pilot assessment of virus particle characteristics after gas plasma exposure, a higher magnification (80kX), for which five virus particles for gas plasma exposure and eight virus particles for untreated controls were analyzed). The majority of MHV particles exhibited a distinct spherical form. The regular diameter of the untreated virus particles (Fig. 7b, left virion) was about 85 nm, and the enveloped nature of MHV was visible in the treated and untreated virions. Interestingly, a qualitative notion was that the gas plasma-exposed MHV envelope membranes and core particle regions appeared more speckled and, in some cases, slightly disintegrated at places (Fig. 7b, right virion) compared to smoothly membraned untreated MHV. In addition, there was the notion that some virus particles tended to have increased membrane envelope diameters in the latter, indicating a potential dilatation of an altered membrane envelope (Fig. 7c).

## 4 Discussion

Murine hepatitis virus (MHV) remains a powerful and widely used coronavirus model for understanding various aspects of the efficacy of antiviral therapies in respiratory disease. Due to similarities with SARS-CoV-2 in genetics, structure, immune response, replication, pathogenesis, and tissue tropism [30], MHV, which allows work at biosafety level 2, has been widely used in coronavirus research [3]. Novel technologies such as gas plasma, which generates various potentially anti-virucidal components, could, in principle, be used to combat respiratory viral infections in the future [31]. Here, we identified potent plasma-induced antiviral activity against murine MHV infectivity in murine 17Cl-1 fibroblasts. We could identify reactive species potentially pivotal in mediating such effects and RNA modification or degradation as possible additional mechanisms of action for longer gas plasma exposure times, along with changes found in the virus particle morphology following gas plasma treatment.

Respiratory diseases are a common and significant cause of morbidity and mortality worldwide and are associated with increasing healthcare costs. Emerging global pandemics, such as severe respiratory syndrome caused by viruses (e.g., COVID-19), require new effective strategies to combat them [16]. Generally, redox stress induced by increased production of reactive oxygen and nitrogen species (ROS) and decreased host antioxidant response contributes to inflammation and the pathogenesis of viral infection [7]. The massive release of inflammatory cytokines after coronaviral infections leads to acute brain injury and substantial changes in the neurotransmitters, including serotonin and calcitonin gene-related peptide [5]. Conversely, ROS provides antimicrobial properties and induces a cascade of cellular and molecular responses [32]. Nrf2 pathway inhibits the expression of pro-inflammatory cytokines and the development of cytokine, showing that Nrf2 activators could be an adjuvant therapeutic strategy for managing severely affected COVID-19 patients [5]. In addition, studies with gas plasma have shown a beneficial effect on modifying Nrf2 signaling and cytokine signatures in preclinical and clinical studies [[33], [34], [35]]. Gas plasma-generated ROS generate new prospects for the effective use of gas plasma, including cleaning biological surfaces (e.g., food, tissues, and wounds) from various microbial pathogens [36,37]. Antibacterial, antifungal, and antiviral effects of gas plasmas have been demonstrated before [[38], [39], [40], [41]], leading to increasing interest in this novel technology. For instance, effective inactivation of non-enveloped bacteriophage MS2 and enveloped airborne porcine reproductive and respiratory syndrome virus (PRRSv) was demonstrated using a packed bed dielectric barrier discharge plasma device [42]. Direct high-voltage atmospheric cold plasma DBD has been used to inactivate Tulane virus on chicken breast [43]. In ophthalmology, a dielectric barrier discharge (DBD) was found to suppress herpes simplex virus (HSV-1) replication in the corneal epithelium [17]. The broad spectrum of successful gas plasma applications and our promising results in controlling MHV particle infectivity underscores the potential of gas plasma technology.

The mechanisms of action of gas plasma-induced antiviral activity are still under debate. Key mediators of antimicrobial gas plasma effects are often reactive species [44], including free oxygen radicals with an unpaired electron such as superoxide anions, hydroxyl radicals (•OH), alkoxyl radicals (RO•), and peroxyl radicals (ROO•), as well as non-radical oxidants such as hydrogen peroxide (H_2_O_2_), singlet delta oxygen, and ozone (O_3_) [44]. Although triplet dioxygen (•O_2_••) and superoxide anion (O_2_^−^•) fall under this definition, their non-radical chemistry dominates their reactivity [45]. The product of nitric oxide (NO) and superoxide (O_2_^−^), peroxynitrite (ONOO^−^) [46], was previously suggested to have antiviral effects (in terms of its chemical production and not the generation of gas plasma processes) in, e.g., hantavirus [11] and coxsackievirus [47]. Gas plasmas produce various reactive molecules simultaneously, challenging their individual assessment and contribution. Scavengers with known activity and specificity for reducing the action of oxidants and radicals can support the evaluation of identifying reactive molecules potentially pivotal in mediating gas plasma-mediated antiviral activity. Glutathione (GSH) is an efficient oxygen radical scavenger that revealed the highest reduction of gas plasma-mediated abrogation of MHV infectivity. Interestingly, a previous report stated that GSH can also have scavenging activity on the ONOO^−^ anion [48]. In addition, ONOO^−^ was previously suggested to modify SARS-CoV-2 spike (S) proteins and, by this, reduce viral infectivity [49]. Also, the amino acids tyrosine and cysteine effectively reduced gas plasma-mediated MHV infectivity. Both scavenge H_2_O_2,_ but results using catalase, which did not effectively protect MHV from gas plasma inactivation, suggest, contrary to findings in cell biology with the kINPen gas plasma exposure [29], a negligible role of H_2_O_2_ as an antiviral agent produced by gas plasmas or cells. Yet, the two amino acids are also known to scavenge superoxide effectively, a product often produced in infected cells but less described for its direct antiviral effects [50]. However, as a reaction partner for ONOO^−^ generation, superoxide could play an indirect role as an antiviral gas plasma-produced agent, as our data suggest. This is seconded by tyrosine also being a scavenger of ONOO^−^ directly [51], supporting this assumption. Both tyrosine and GSH also readily react with OH•. Yet, since other OH• scavengers such as mannitol and DMSO only modestly protected MHV from gas plasma inactivation, this radical might play a lesser role as a central mediator. Along similar lines, GSH readily reacts with singlet delta oxygen. Yet, the singlet delta oxygen and OH• scavenger histidine only provided 50 % of the viral protection from gas plasma modification, as seen with GSH. Moreover, ascorbic acid has been shown to react with OH•, H_2_O_2_, and O_2_^−^•, regenerating α-tocopherol from tocopheroxyl radicals, thereby protecting the membranes, including those surrounding MHV, from oxidative damage [52]. Hence, we conclude that the weak oxidant H_2_O_2_ [53] does not cause antiviral effects in gas plasma-treated MHV, while the effects observed may be due to the simultaneous action of several reactive species types, especially peroxynitrite and also hydroxyl radicals and singlet oxygen, with varying degrees of contribution to antiviral effects. Identifying the relative potency of different reactive species types, supported through gas plasma research, may also enable novel future antiviral treatment options based on using specific reactive agents matched to viruses found to be sensitive against the same.

In general, several mechanisms have been proposed for virus inactivation through gas plasmas. First, simulations and specific experiments showed an interaction of ROS with head groups of the lipid bilayer by their oxidation, resulting in a slight initial increase in membrane rigidity and a substantial increase in fluidity [54,55]. The resulting decrease in lipid assembly could allow ROS to enter the virus's interior and weaken its ability to enter the cell in the first place. Secondly, posttranslational modifications of proteins (oxPMTs) [56,57], peptides [58,59], amino acids [60], and lipids [61,62] caused by ROS have been found. While MHV is a single-stranded RNA virus with an outer membrane containing structural proteins, such as spike (S), envelope (E), nucleocapsid (N), and membrane (M) protein, we verified an average size of about 85 nm by electron microscopy, which was similar to the size of cryo-fixed MHV virions (86 nm) [63]. Structural and functional changes (e.g., viral M protein) of MHV with oxPTMs may lead to a reduction of particle load and reduced viral entry into host cells. Iron oxide nanozyms can catalyze lipid peroxidation of the viral lipid envelope to inactivate influenza virus as shown before, ultimately providing protection against viral transmission and infection [64]. Moreover, our study revealed a reduction of intact MHV RNA following gas plasma exposure, especially for extending treatment times. This may be a consequence of gas plasma-derived reactive species reaching the nucleic acids through the membrane and capsid and, more likely, by viral RNA being released through oxidative membrane and capsid destruction being freely exposed to gas plasma-derived reactive species in the liquid.

Our study faces challenges. First, the murine coronavirus (MHV) may not fully replicate the pathogenesis of SARS-CoV-2 in humans. One of the most important limitations is the difference in receptor usage between MHV and SARS-CoV-2. MHV primarily uses CEACAM (carcinoembryonic antigen-related cell adhesion molecule) 1 as its receptor for cell entry [65], whereas SARS-CoV-2 uses angiotensin-converting enzyme (ACE) 2 [66]. Hence, MHV cannot model the interaction between SARS-CoV-2 and its human receptor, which is critical for understanding viral entry and developing targeted therapies. Although there are similarities in immune responses and tissue tropism, human coronavirus disease was found to be influenced by additional factors, such as co-morbidities, which may not be adequately captured in MHV models. Second, due to the lack of relevant preclinical or clinical viral models, we investigated gas plasma-induced effects only in liquids containing MHV. Translational applications require, however, either virus reduction in or on the epithelium in medicine or on surfaces in the fields of medicine and hygiene, respectively. Third, regarding medical applications, the penetration depth of gas plasma-derived ROS is still under debate and likely dependent on various factors that are not recapitulated by our virus suspension treatment.

## 5 Conclusion

We are the first to describe the antiviral effect of an atmospheric pressure plasma jet approved for medical use and routinely used in clinical practice. Gas plasma exposure effectively inactivated murine coronaviruses and reduced their infectivity in fibroblasts. We identified some types of reactive species likely to be critical in mediating such effects, such as ONOO^−^, while others, such as H_2_O_2_, are likely to be less relevant in the mechanism of action. In addition, we demonstrated a moderate contribution of ROS-mediated RNA modification or degradation to the observed antiviral gas plasma effects. At the same time, morphological changes of gas plasma-treated virions were found, and envelope disruption may cause gas plasma-mediated reduction of MHV infectivity. The results may be helpful for future antiviral gas plasma applications by shedding new light on potentially novel ways to directly use reactive species as antiviral agents.

## CRediT authorship contribution statement

**Sander Bekeschus:** Conceptualization, Methodology, Software, Validation, Formal analysis, Resources, Data curation, Writing C original draft, Writing – review & editing, Visualization, Supervision, Project administration, Funding acquisition. **Meike Heuser:** Software, Validation, Formal analysis, Investigation, Data curation, Writing – review & editing, Visualization. **Lea Miebach:** Methodology, Validation, Investigation, Writing – review & editing. **Marcus Frank:** Methodology, Software, Formal analysis, Investigation, Writing – review & editing. **Thomas von Woedtke:** Writing – review & editing, Funding acquisition. **Anke Schmidt:** Methodology, Formal analysis, Investigation, Writing – original draft, Writing – review & editing, Visualization, Supervision.

## Funding

This work was funded by the German 10.13039/501100002347Federal Ministry of Education and Research (BMBF), grant numbers 03Z22DN11, 03Z22Di1, and 03COV06A. The funding source had no role in the design of this study or its execution, analyses, interpretation of the data, or decision to publish results.

## Declaration of competing interest

The authors declare no conflict of interest with regard to publication of this manuscript.

## Data Availability

Data will be made available on request.
