# Supplementary figures and images for: Viral inactivation of murine coronavirus via multiple gas plasma-derived reactive species
Source: Redox Biol. 2025 Mar 10;82:103591. doi: 10.1016/j.redox.2025.103591 (PMC11954120; doi:10.1016/j.redox.2025.103591)

## Supplemental Figure S1


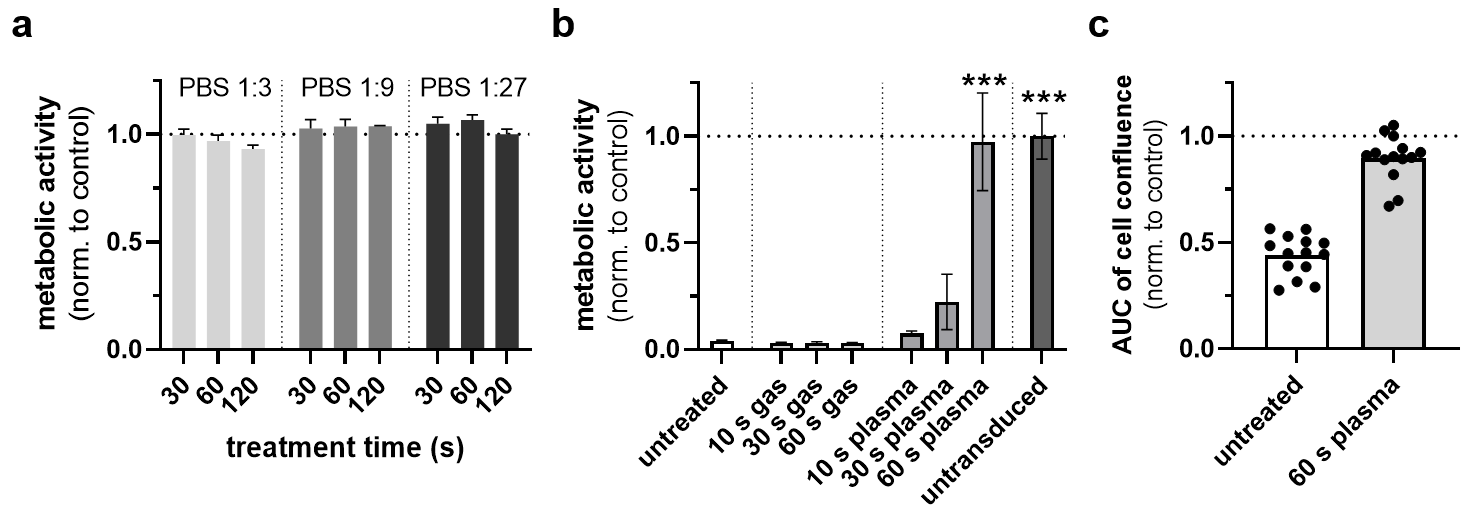


## Supplemental Figure S2


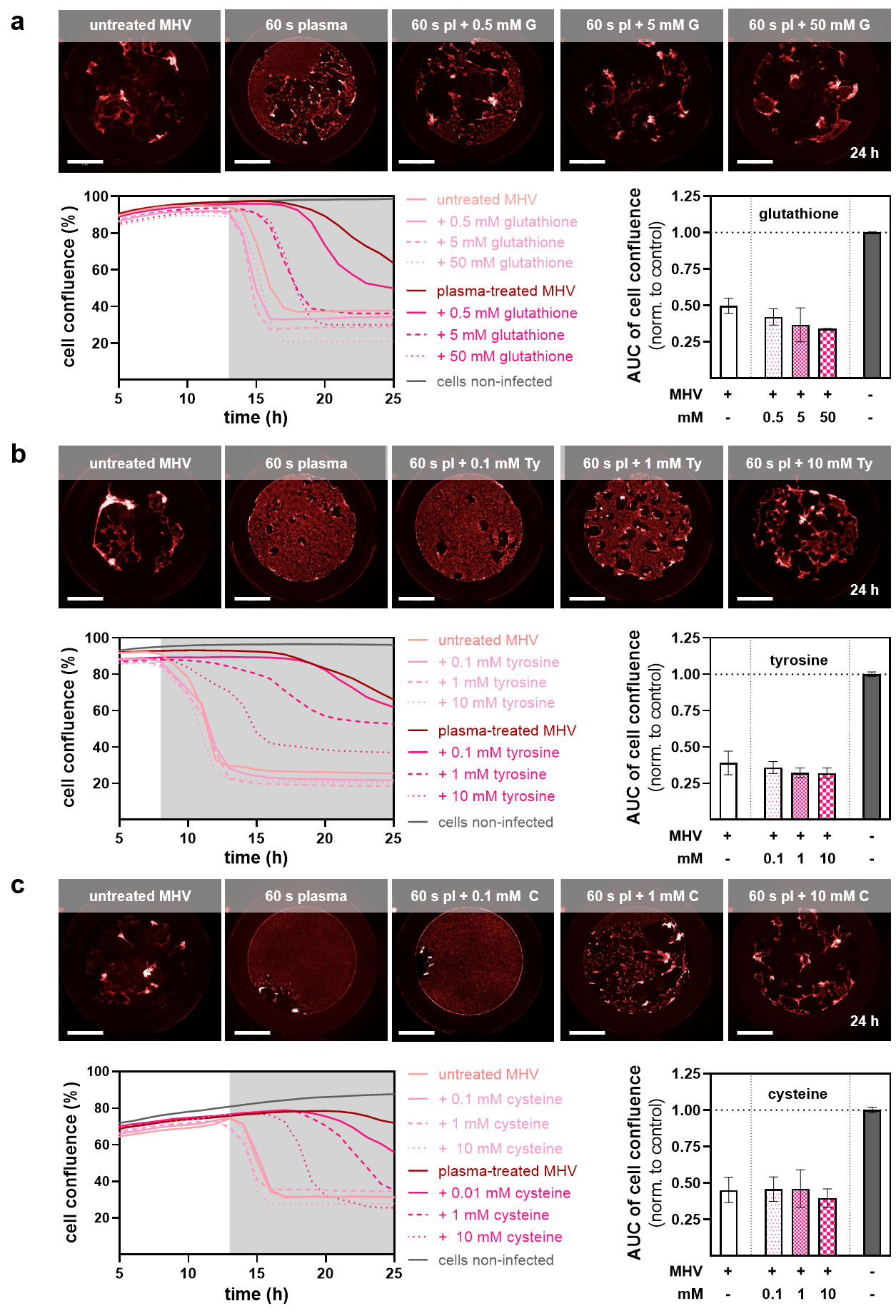


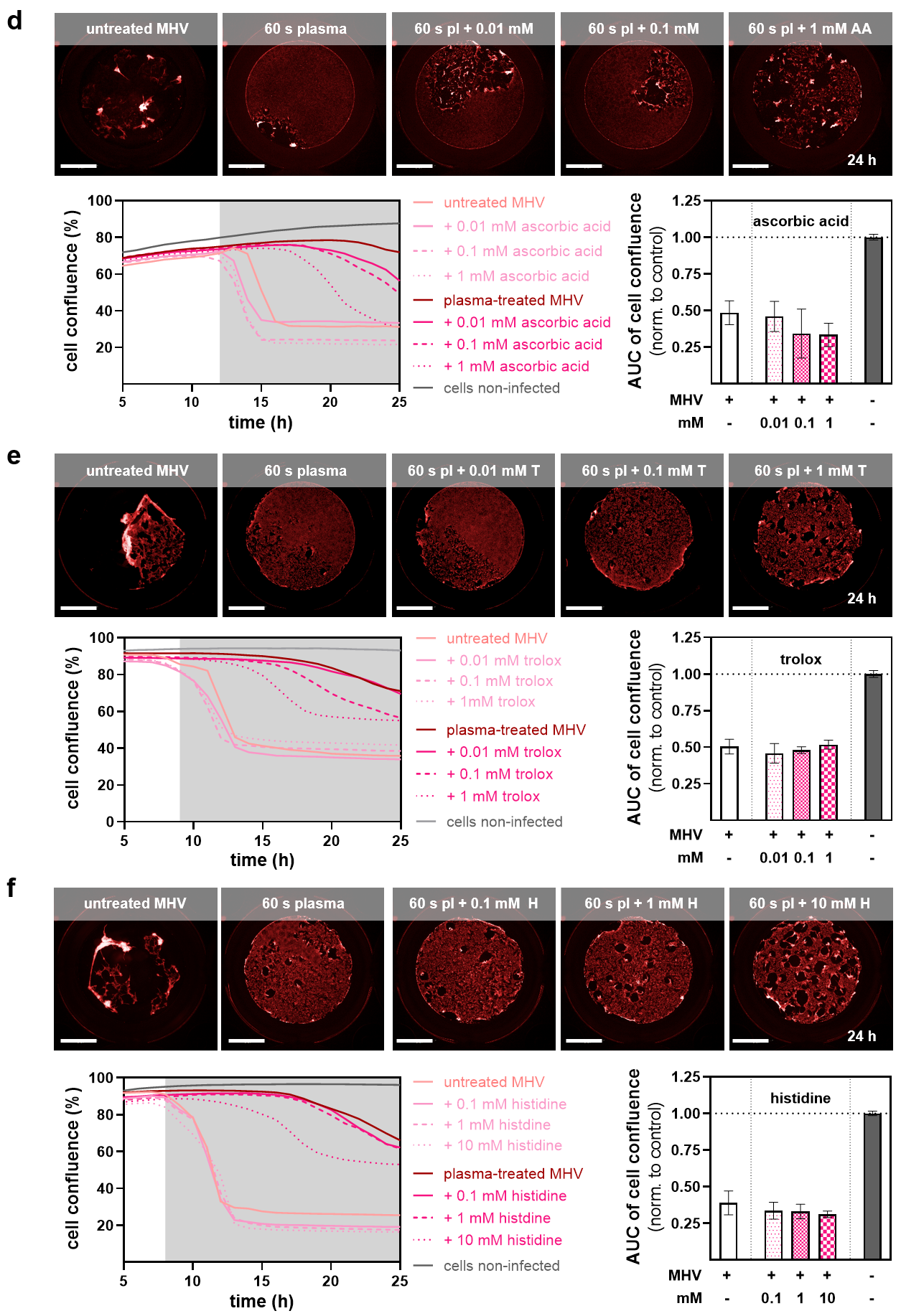


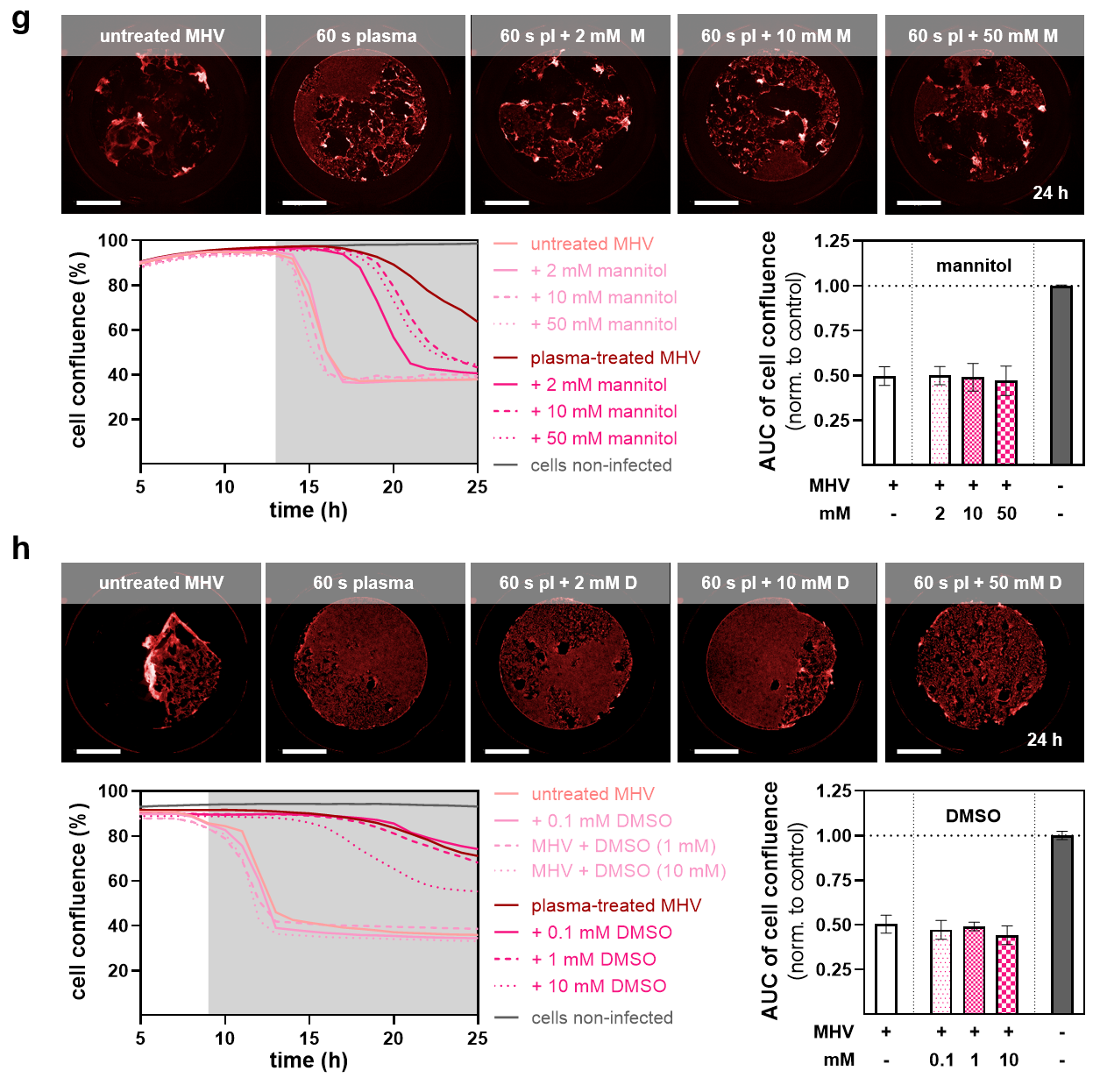


## Supplemental Figure S3


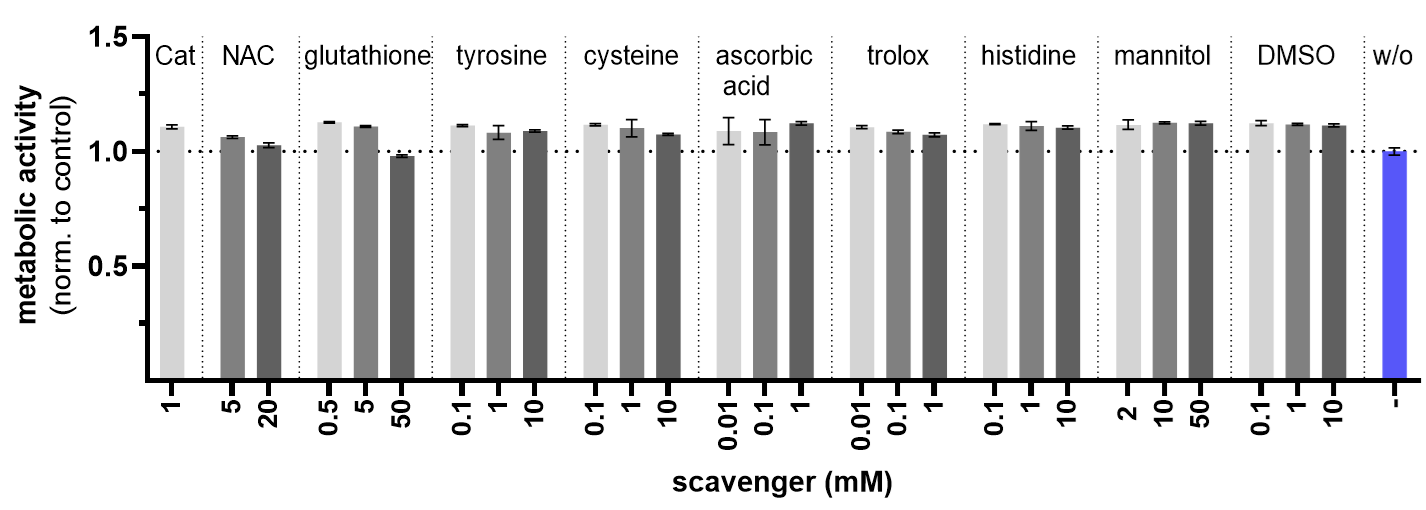


## Supplemental Figure S4


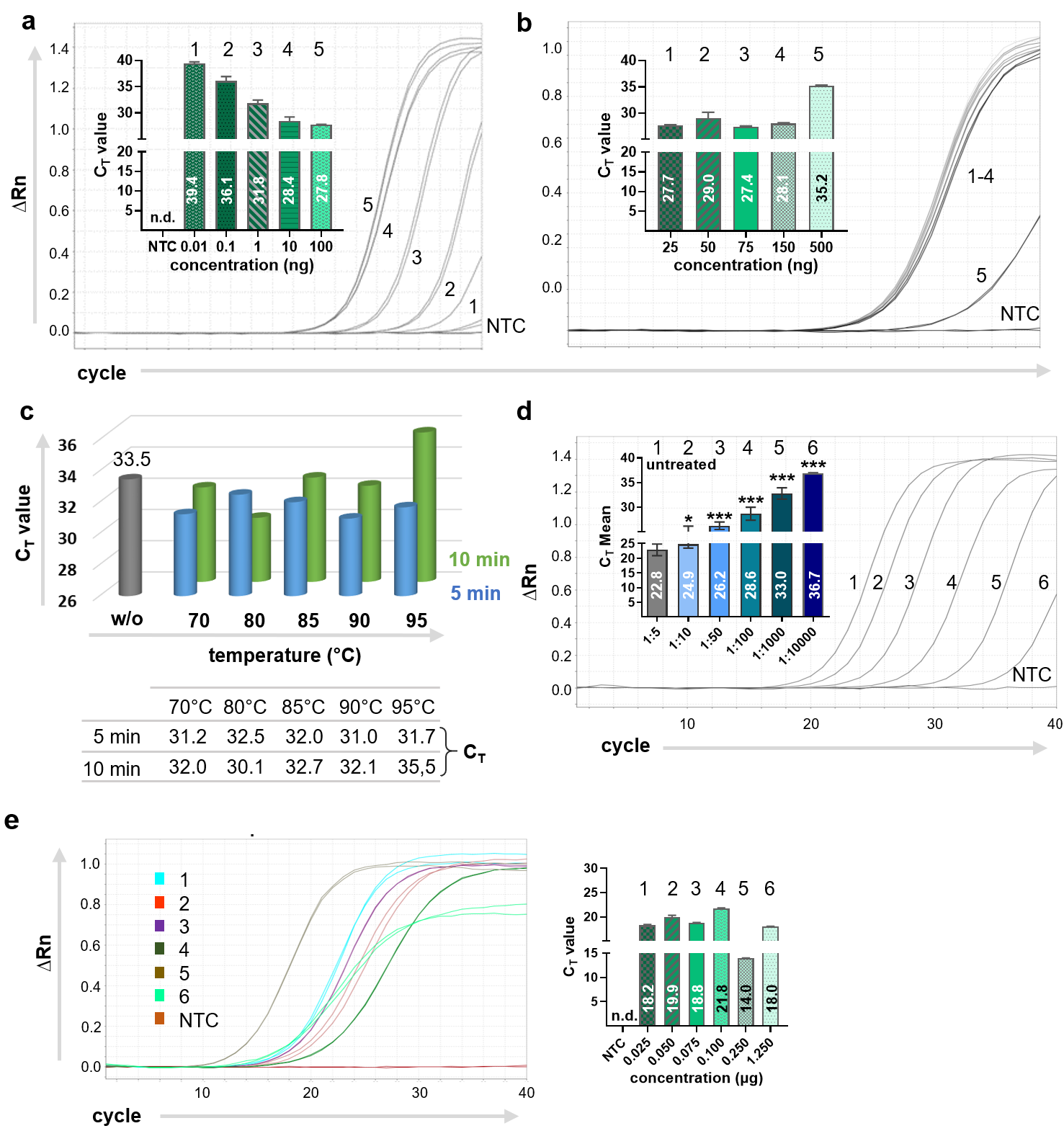

Supplement: Multimedia component 1 [file mmc1.docx]
